# Behavioral Patterns Toward Preventive Dental Care at School Dental Camps: An Observational Study in Bengaluru

**DOI:** 10.7759/cureus.54294

**Published:** 2024-02-16

**Authors:** Rohan Shinkre, Siya Dukle, Ishan Mukherji, Aarya Bharadwaj, Rashmi Naik, Nikhil V Suresh, Sneha Jaiprakash Pednekar, Shruthi Eshwar, Srivastava B K

**Affiliations:** 1 Central Research Wing, KLE Society's Institute of Dental Sciences, Bengaluru, IND; 2 Department of Pediatric and Preventive Dentistry, Goa Dental College and Hospital, Bambolim, IND; 3 Department of Public Health Dentistry, Guru Nanak Institute of Dental Sciences and Research, Kolkata, IND; 4 Department of Public Health Dentistry, KLE Society's Institute of Dental Sciences, Bengaluru, IND; 5 Department of Public Health Dentistry, KLE Society’s Institute of Dental Sciences, Bengaluru, IND

**Keywords:** school camp, mobile dental van, dental fear, dental anxiety, behaviour management problems

## Abstract

Background

Dental behavior management problems of children towards preventive dental care at school dental camps in India remain largely undocumented. This study aimed to assess such behavior patterns in preschool and school-age children at a school dental health camp.

Materials and methods

The cross-sectional study included 462 children, with 261 children each in the preschool (three to five years old) and school (six to 12 years old) age groups in Bengaluru. On the school dental camp day, their behavior and anxiety were gauged using the Frankl Behavior Rating Scale and the Raghavendra, Madhuri, and Sujata Pictorial Scale, respectively. The Chi-square test was used to uncover predictive variables for children's behavior patterns toward preventive dental procedures at the dental school camps.

Results

A high prevalence of definitely negative Frankl Behavior Rating Scale ratings (59%, n=272) and dental anxiety (53%, n=245) were noted among the participants. Age, sex, the area of residence of the child, and the previous history of dental visits and treatment were predictors of their behavior at a school dental camp setup.

Conclusion

The present study gives an insight into the behavior of children towards preventive dental care at a school dental camp in a mobile dental van, stressing the need for behavior assessment before the treatment.

## Introduction

Dental fear is a severe issue for dentists, as it usually results in various dental behavior management problems (DBMP). Dental fear has a complex etiology that is still not fully understood. Age, the conduct of parents, parental anxiousness, prior medical and dental history, type of dental environment, dread of pain, apprehension of strange places and people, being apart from their parents, feeling out of control, and the procedures themselves can all have an impact on a child's conduct during a dentist appointment and contribute to fear of dentistry [1,2]. The pooled* *prevalence of dental fear globally in preschoolers, school children, and adolescents was found to be 36.5%, 25.8%, and 13.3%, respectively, with more predilection in the former two age groups as opposed to the latter [3].

The kind and caliber of care children receive is greatly influenced by how they react to dental treatment. Hence, a wide array of robust socio-emotional and behavioral rating scales have been developed to assist dentists in examining and evaluating the child's psychological and behavioral responses, which aids in tailoring a prudent behavior guidance method that promotes dental care in a clinic and offers a way to systematically document behaviors for subsequent sessions [4].

In India, mobile dental van (MDV) programs are a component of dental initiatives planned and carried out by organizations and dental institutes, especially those that include post-graduation in public health dentistry in their curricula. MDVs are utilized through dental camps to offer dental care to disadvantaged, uninsured, and rural communities. These vans contain fully furnished dental operatories, a waiting area, and an educational space. They address the geographical, temporal, and cultural constraints on health care delivery in a developing nation with an enormous population, like India. The dentistry camps are frequently a one-day visit to rural or distant places or a school environment to provide treatments, particularly preventive care, disease screening, and health education [5]. As a result, the environment at a dental camp is different from a conventional clinical setting and presents a unique set of difficulties.

There is a pronounced deficit in documentingdental behavior problemstoward preventive dental care in MDVs in a school dental camp setting among preschool and school children. The need for this study raises the research gap, which can be instrumental in designing better strategies to facilitate adequate handling of dental anxiety and dread and to optimize the delivery of basic oral health care services in such camp settings. 

## Materials and methods

Study setting and population

This cross-sectional study included preschool (three to five years) and school-age (six to 12 years) children and was carried out in Bengaluru, India, from February 1, 2022, to March 31, 2022, following the Strengthening the Reporting of Observational Studies in Epidemiology (STROBE) guidelines. Before the study, ethical approval was obtained for the study from the Institutional Review Board of the KLE Society's Institute of Dental Sciences, Bengaluru (REF/IRB/CODE-KLE/JAN2022/13). Informed written consent in the local Kannada language was obtained from each school before the study. The school was also instructed to obtain the same from the parent or guardian of the child before the school camp day.

Sample size estimation

Assuming a 50% prevalence of the negative behavior of children toward preventive dental procedures at a school dental camp set-up**,** with a confidence level of 95% and a margin of error of 0.05, the minimum sample size was determined to be 385. Assuming contingencies of 20%, it was inflated to 462. Hence, the required sample size for each of the two age groups of preschool and school-going children was 261, respectively.

Study instruments

Data about demographic characteristics was collected from the school registries while the children were interviewed to assess their previous dental visit history. Two validated scales were selected to assess the behavioral tendencies toward the dental treatment that were assessed by the investigators during the treatment.

Frankl's Behavior Rating Scale (FBRS) was used to examine the child's behavior. It is symptomatic of dental fear because children with negative behavior toward dental procedures are more likely to exhibit fear of such procedures​​​​​​​.It categorizes a child's behavior into four categories based on the child's demeanor while receiving dental care, namely: definitely negative (refusal of treatment, crying forcefully, fearful, or any other overt evidence of extreme negativism), negative (reluctant to accept treatment, uncooperative, some evidence of negative attitude but not pronounced, sullen, withdrawn), positive (acceptance of treatment, at times cautious, willingness to comply with the dentist, at times with reservation, but patient follows the dentist's directions cooperatively), and definitely positive (good rapport with the dentist, interested in the dental procedures, laughing and enjoying) [6], as assigned by the clinician. The ratings were given as per the following characteristics perceived in the child:

Raghavendra, Madhuri, and Sujata Pictorial Scale (RMS-PS) was used to assess dental anxiety among the children in both groups. It is a self-reported scale that consists of five faces in a row ranging from extremely cheerful to dissatisfied. For boys and girls, two different sets of photos are used, and the children are instructed to select the expression they liked best about themselves at the time. A very pleased look was coded as 1, and a very unhappy face was coded as 5 [7].

Training and calibration

A pilot study was done for the training and calibration of the examiners on the assessment of behavior at a school dental camp using Frankl's scale. It was done on children randomly selected from one preschool and school from the list of schools allotted. In this pilot study, the aggregate number of children was 10% of the projected sample size, which amounted to 46 (23 in the preschool and school-age range, respectively). The two investigators (Rohan Shinkre and Aarya Bharadwaj) were trained by an experienced pedodontist to assess the children's behavior in both groups using the FBRS. The Kappa statistics for their inter-rater reliability were calculated to be excellent, with a value of 0.92 at a 95 % confidence interval. No inter-rater reliability was calculated for the RMS-PS as it was a self-reported scale. The pilot study participants were not incorporated into the final study.

Data collection

A simple random sampling was employed, yielding two preschools and two schools (one public and one private) from Bengaluru that were selected from the list of schools obtained from the office of the Deputy Director of Public Instruction (DDPI), Bengaluru using the lottery method. In the second stage, students who satisfied the selection parameters were chosen from these schools with the help of a randomly generated sequence of their allotted roll numbers till the desired sample size was met.

Preschool and school children aged three to 12 years were enrolled in the study that required preventive treatment that included topical fluoride applications and pit and fissure sealants in the MDV at the dental camp. Those with no parental/guardian consent or with any pain-inducing oral pathology, medically compromised children, and those with physical, mental, and intellectual disabilities were excluded.

Statistical analysis

Descriptive statistics such as mean and standard deviation were computed for continuous data, and frequencies and percentages for categorical data. The association between a child's behavior and related factors was assessed using the chi-square test. The significance of the statistical analysis was evaluated at a 5% level of significance using SPSS version 26.0 (IBM Inc., Armonk, USA).

## Results

**Figure 1:**
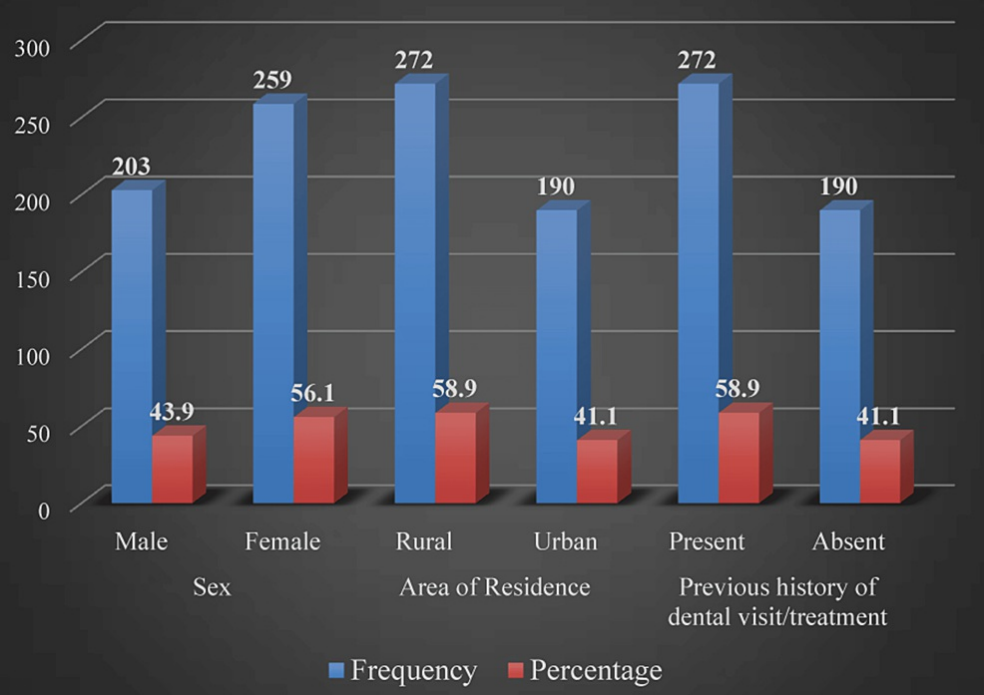
Demographic characteristics of study participants

The study incorporated 462 children, with a mean age of 6.22±2.32 years. Overall, more than 50% of the participants were females (n=259, 56.1%), living in rural areas (n=272, 58.9%), and having visited a dentist or been treated by them previously (n=272, 58.9%; see Figure 1).

**Figure 2:**
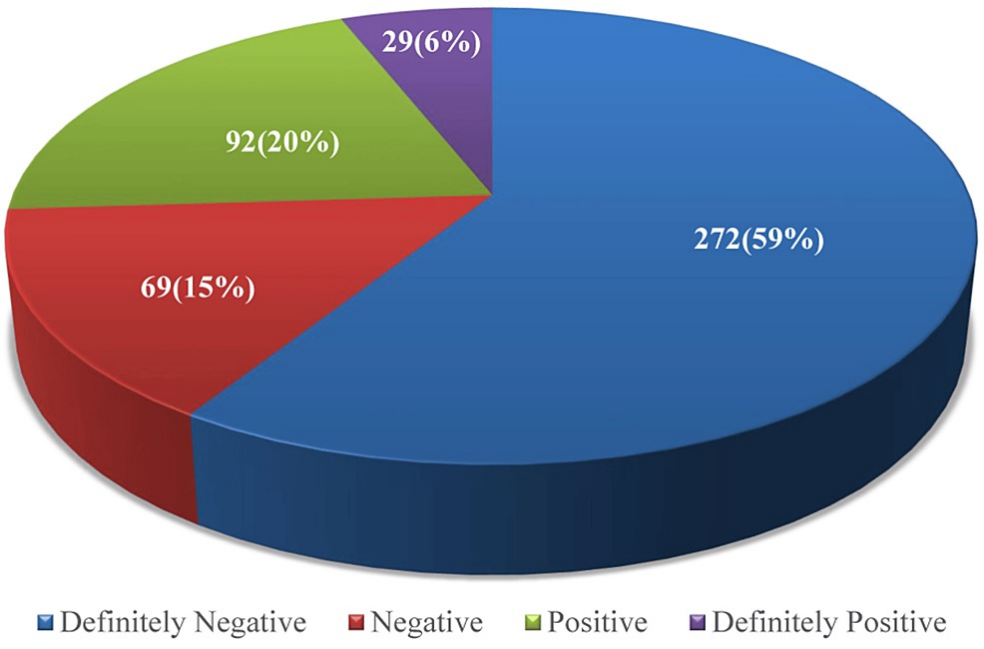
Distribution of study participants based on behavior using Frankl's Behavior Rating Scale

**Figure 3:**
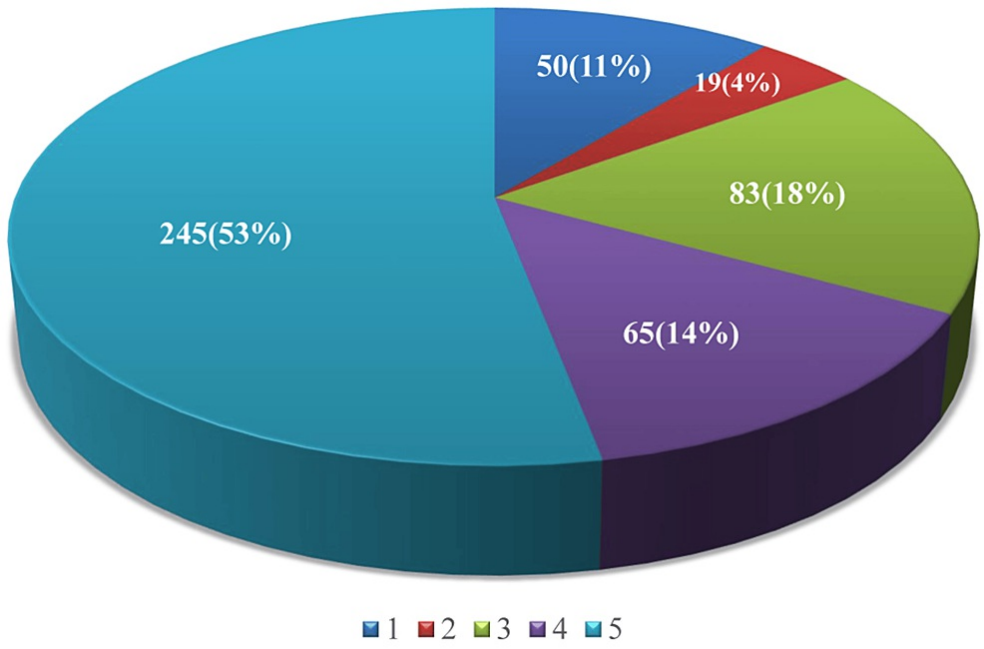
Distribution of study participants based on dental anxiety using the Raghavendra, Madhuri, and Sujata Pictorial Scale Scores 1 to 5 represent the level of dental anxiety among the children, where 1 shows the least and 5 the highest level of dental anxiety measured by the Raghavendra, Madhuri, and Sujata Pictoral Scale.

The behavior and anxiety among study participants were in 59% (n=272) of participants definitely negative, only 6% (n=29) were definitely positive, and 53% (n=245) chose the most unhappy face on the RMS-PS indicating high levels of dental anxiety, while 11% (n=50) were less anxious about the dental intervention (Figures 2-3).

The factors associated with study participants' behavior were age, sex, area of residence, and previous dental exposure, all of which showed a statistically significant association (p<0.05). More than half, i.e., 68% of preschool children, 67.5% of males, and 64% of children living in a rural area, had definitely negative behavior as compared to 49.8% among school-age children, 52.1% among females and 51.6% of children in urban areas respectively. However, 64.3% of children with previous dental exposure had definitely positive behavior, as compared to 51.1% who had never visited the dentist or previously undergone dental care (Table 1).

**Table 1:** Factors associated with children's behavior in a school dental camp setting

Independent variables			Frankl's Behavior Rating Scale				χ^2 ^statistic	p-value
			Definitely negative	Negative	Positive	Definitely positive		
Age groups	School-age	N	115	44	47	25	26.97	<0.01
		%	49.8%	19%	20.3%	10.8%		
	Preschool age	N	157	25	45	4		
		%	68%	10.8%	19.5%	1.7%		
Sex	Male	N	137	20	39	7	15.53	0.01
		%	67.5%	9.9%	19.2%	3.4%		
	Female	N	135	49	53	22		
		%	52.1%	18.9%	20.5%	8.5%		
Area of residence	Rural	N	174	34	51	13	8.36	0.04
		%	64%	12.5%	18.8%	4.8%		
	Urban	N	98	35	41	16		
		%	51.6%	18.4%	21.6%	8.4%		
Previous history of dental visit/treatment	Present	N	34	51	12	175	10.09	0.02
		%	12.5%	18.8%	4.4%	64.3%		
	Absent	N	35	41	17	97		
		%	18.4%	21.6%	8.9%	51.1%		

The factors associated with the study participants' dental anxiety were age and sex. More than half, i.e., 58.9% of preschool children and 60.6% of males, had the highest level of dental anxiety as compared to 47.2% of school-age children and 47.1% of females, respectively (Table 2).

**Table 2:** Factors associated with children's dental anxiety in a school dental camp setting Scores 1 to 5 represent the level of dental anxiety among the children, where 1 shows the least and 5 the highest level of dental anxiety measured by the Raghavendra, Madhuri, and Sujata Pictoral Scale.

Independent variables			Raghavendra, Madhuri, and Sujata Pictorial Scale					χ^2 ^statistic	p-value
			1	2	3	4	5		
Age groups	School-age	N	32	15	34	41	109	20.42	<0.01
		%	13.9%	6.5%	14.7%	17.7%	47.2%		
	Preschool age	N	18	4	49	24	136		
		%	7.8%	1.7%	21.2%	10.4%	58.9%		
Sex	Male	N	16	5	38	21	123	12.88	0.01
		%	7.9%	2.5%	18.7%	10.3%	60.6%		
	Female	N	34	14	45	44	122		
		%	13.1%	5.4%	17.4%	17%	47.1%		
Area of residence	Rural	N	26	9	50	30	157	9.2	0.06
		%	9.6%	3.3%	18.4%	11%	57.7%		
	Urban	N	24	10	33	35	88		
		%	12.6%	5.3%	17.4%	18.4%	46.3%		
Previous history of dental visit/treatment	Present	N	25	9	50	31	157	8.83	0.07
		%	9.2%	3.3%	18.4%	11.4%	57.7%		
	Absent	N	25	10	33	34	88		
		%	13.2%	5.3%	17.4%	17.9%	46.3%		

## Discussion

The current study aimed to investigate preschool and school children's behavior patterns and influencing factors toward preventive dental treatment in dental school camp outreach programs. In the present study, we found a high prevalence of definitely negative behavior based on Frankl's Behavior Rating Scale ratings and dental anxiety among the study participants. Similar high levels of dental fear and anxiety were detected by Kumar et al. in children of similar age in a hospital setting in a South Indian population [8].

Our findings suggest that age predicts behavior and dental anxiety in a school dental camp setting. Compared to school-aged children, more pre-schoolers rated negatively on the FBRS and experienced dental anxiety. The results of earlier studies correspond with our findings [6,9]. The study conducted by Baier et al. showed that children under the age of six were comparatively more likely to display negative behaviors in a dental setting [6]. This may be due to their limited intellectual and cognitive development, as a result of which the younger children are unable to fully grasp the nature of dental therapy, realistically assess it, or comprehend the explanations and directions. It can also be ascribed to the notion that additional years in school equip children to follow directions and engage in unfamiliar activities [10,11].

Boys scored higher for negative behavior and dental anxiety than girls. There is conflicting information regarding the sex variations in dental fear and anxiety. These findings could be explained by several variables, including the study population's cultural background, the construct of the anxiety scales employed, and the actual distinctions in anxiety across sexes or amalgamations of these variables [10,12].

Children with rural backgrounds were less cooperative with the treatment procedures than their urban counterparts. A study on adolescents discovered that individuals from rural areas tended to be more apprehensive about getting dental work than those from metropolitan areas [13]. This could be in part due to a dearth of awareness and a lesser proclivity to attend a hospital or clinic as a result of inadequate access to basic healthcare services [14].

Compared to individuals who had never had contact with a dental health care professional, those with a history of dental visits or treatment had a more favorable attitude about dental therapy. This might be due to the children growing older and more mature with each dental appointment and earlier dental experiences fostering confidence between the patient and the dentist [10].

With a population of over a billion, India is a sizable nation with health disparities. In India, MDVs are utilized to help deliver primary oral healthcare services. Compared to stationary dental clinics, mobile dental clinics provide medical care to underserved communities in impoverished urban and remote areas. Children from low socioeconomic homes have high no-show rates, dental caries and other oral conditions, limited access to treatment, and a lack of transportation. Therefore, these school-based mobile dentistry clinics become a means to provide basic dental care for the children who live in such hard-to-reach places [15,16].

This novice investigation was needed to gauge the burden of dental dread and anxiety in a school dental camp setup, which was largely uncharted territory. Since MDVs are a viable outreach method for accessing the target population of preschool and school-age children, understanding their behavior in camp settings is the key to optimal healthcare delivery in these underserved areas to diminish the disparities in healthcare. This study also emphasizes the necessity to effectively train public health dentists to identify and address these behavioral tendencies among children during such camps.

The present study had certain limitations. The cross-sectional design of the study hinders the determination of the temporality of associations of the predictor variables uncovered in the study with these behavior patterns of children at the school dental camps. The parental influences and the socioeconomic factors that have been shown to influence dental behavior in a stationary clinical setup could not be incorporated in the present study as access to the parents in the school premises during school hours was not feasible.

## Conclusions

Within the limitations, this study gives an insight into the behavioral patterns observed in preschool and school children towards preventive treatment in an MDV. It shows a positive association of a child's behavior in an MDV towards preventive dental care at school camps with age, sex, the area of residence of the child, and the previous history of dental visits and treatment, thereby providing sufficient input to the factors that must be identified and addressed to guarantee better reception of such dental care in a camp setting.
